# The value of frozen section diagnosis of tumor spread through air spaces in small-sized (≤ 2 cm) non-small cell lung cancer

**DOI:** 10.1186/s12957-023-03092-9

**Published:** 2023-07-03

**Authors:** Yun Ding, Shutong Zhao, Xin Liu, Jie Ren, Jiuzhen Li, Weiran Zhang, Meilin Xu, Daqiang Sun

**Affiliations:** 1 Clinical School of Thoracic, Tianjin Medical University, Tianjin, China; 2 Department of Pathology, Tianjin Chest Hospital (Affiliated Hospital of Tianjin University), No. 261, Taierzhuang South Road, Jinnan District, Tianjin, 300222 China; 3 Department of Thoracic Surgery, Tianjin Jinnan Hospital, Tianjin, China; 4 Department of Thoracic Surgery, Tianjin Chest Hospital (Affiliated Hospital of Tianjin University), No. 261, Taierzhuang South Road, Jinnan District, Tianjin, 300222 China

**Keywords:** Tumor spread through air spaces, Small-sized NSCLC, Frozen section Accuracy, Prognosis

## Abstract

**Background:**

The current accuracy of frozen section diagnosis of tumor spread through air spaces (STAS) in non-small cell lung cancer (NSCLC) is poor. However, the accuracy and prognostic value of STAS assessment on frozen sections in small-sized NSCLC (diameter ≤ 2 cm) is unknown.

**Methods:**

Three hundred fifty-two patients with clinical stage I NSCLC (≤ 2 cm) were included, of which the paraffin sections and frozen sections were reviewed. The accuracy of STAS diagnosis in frozen sections was assessed using paraffin sections as the gold standard. The relationship between STAS on frozen sections and prognosis was assessed by the Kaplan–Meier method and log-rank tests.

**Results:**

STAS on frozen sections in 58 of 352 patients could not be evaluated. In the other 294 patients, 36.39% (107/294) was STAS-positive on paraffin sections and 29.59% (87/294) on frozen sections. The accuracy of frozen section diagnosis of STAS was 74.14% (218/294), sensitivity was 55.14% (59/107), specificity was 85.02% (159/187) and agreement was moderate (*K* = 0.418). In subgroup analysis, the *Kappa* values for frozen section diagnosis of STAS in the consolidation-to-tumor ratio (CTR) ≤ 0.5 group and CTR > 0.5 group were 0.368, 0.415, respectively. In survival analysis, STAS-positive frozen sections were associated with worse recurrence-free survival in the CTR > 0.5 group (*P* < 0.05).

**Conclusions:**

The moderate accuracy and prognostic significance of frozen section diagnosis of STAS in clinical stage I NSCLC (≤ 2 cm in diameter; CTR > 0.5) suggests that frozen section assessment of STAS can be applied to the treatment strategy of small-sized NSCLC with CTR > 0.5.

## Background

Lung cancer is a common type of malignancy, with non-small cell lung cancer (NSCLC) accounting for the vast majority of lung cancers [1]. In clinical practice, the prognosis of NSCLC patients is influenced by a number of factors of which tumor spread through air spaces (STAS) is a histological prognostic factor that has not previously been adequately considered previously [2]. In 2015, the World Health Organization (WHO) formally introduced STAS in the classification of lung cancer, defined as clusters of micropapillae, solid nests and/or individual cancer cells spreading into the alveolar space beyond the edge of main tumor [3]. In 2021, the WHO clearly states that STAS is a histological feature with prognostic significance [2]. Several studies have shown that STAS is also an important risk factor for postoperative recurrence of stage I NSCLC [4–6]. In addition, a recent meta-analysis suggested that patients with STAS-positive stage I NSCLC undergoing lobectomy had a better prognosis than those undergoing sublobectomy [7]. For this reason, many surgeons recommend lobectomy for patients with STAS-positive stage I NSCLC [8, 9]. However, this relies on accurate identification of STAS intraoperatively.

As an important method for rapid intraoperative assessment of the benignity or malignancy and histological type of pulmonary nodules, intraoperative frozen section is one of the main tools for guiding surgical strategies for pulmonary nodules. Although the high accuracy of frozen sections in diagnosing the nature of pulmonary nodules, their current accuracy in assessing STAS is poor, with an overall sensitivity of only 44%-54% and a specificity of 80%-91% [10–13]. However, the accuracy of intraoperative frozen section diagnosis of STAS in small-sized (tumor diameter ≤ 2 cm) pulmonary nodules has not been reported. Due to the size limitations of the frozen section, smaller sized nodules may have more alveolar spaces beyond the margin of the main tumor in the frozen section than other larger nodules to assess STAS. Also, ≤ 2 cm in diameter is an indication for sublobectomy in the NCCN guidelines [14], and the results of the JCOG0802/WJOG4607L study support this view [15]. Therefore, we should evaluate the role of frozen section assessment of STAS in small-sized clinical stage I NSCLC with a view to accurate identification of high-risk populations with poor prognoses and decision for the suitable treatment option.

The purpose of this study was to assess the diagnostic accuracy of intraoperative frozen section diagnosis of STAS in small-sized clinical stage I NSCLC (tumor diameter ≤ 2 cm) and to provide a reference for the choice of treatment modality for stage I NSCLC.

## Materials and methods

### Study population

**Fig. 1:**
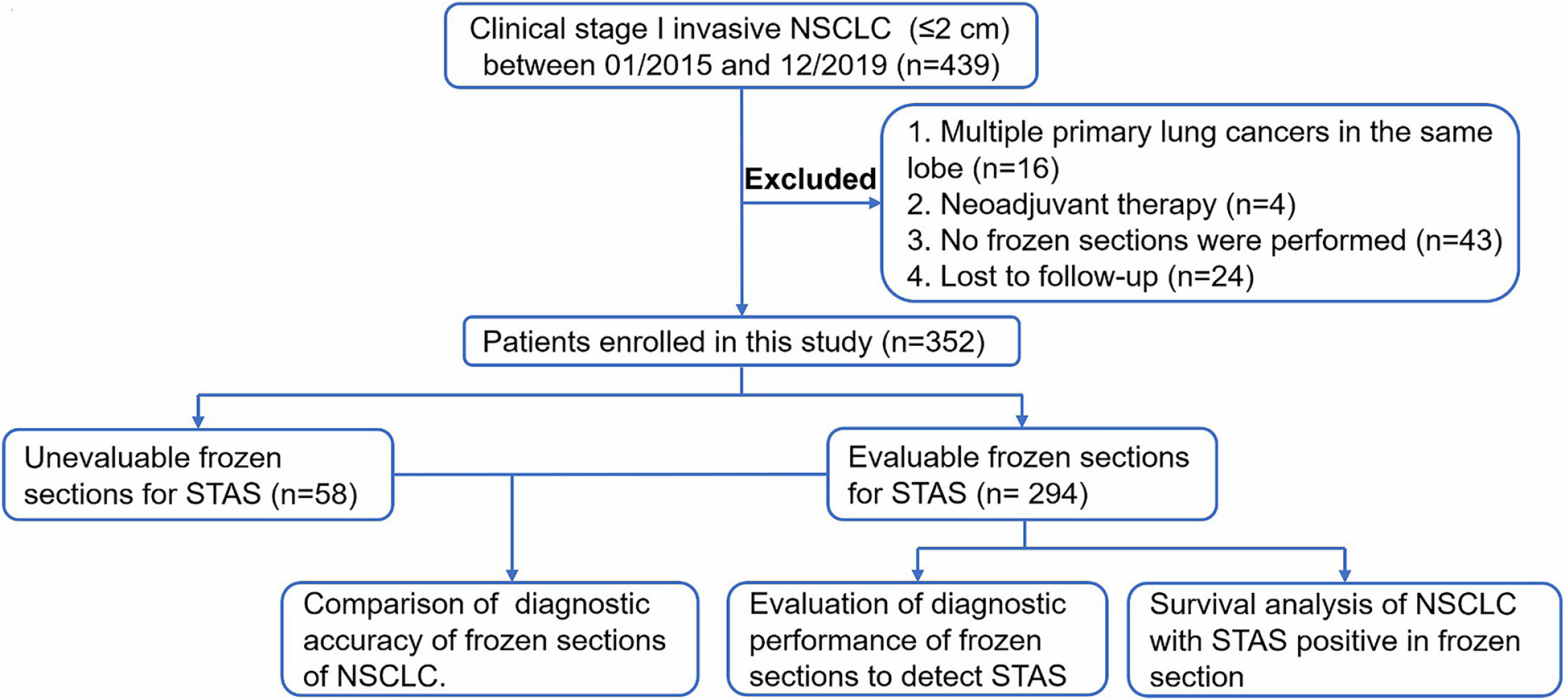
Study design and patient enrolment flow chart

We reviewed the clinical data of patients who underwent surgical treatment for lung cancer at Tianjin Chest Hospital and Tianjin Jinnan Hospital between January 2015 to December 2019 from the hospital databases. The following inclusion criteria were used: (1) clinical stage I NSCLC with tumor diameter ≤ 2 cm based on preoperative imaging diagnosis; (2) underwent complete resection (R0) of pulmonary nodules and postoperative pathologically confirmed invasive NSCLC. The exclusion criteria were as follows: (1) multiple primary lung cancers in the same lobe; (2) preoperative neoadjuvant therapy; (3) no intraoperative frozen sections; (4) loss to follow-up after surgery. The clinical staging was based on the 8th edition of the American Joint Committee on Cancer TNM staging. A total of 352 cases were screened for inclusion in this study, of which 58 cases were not assessed for STAS on frozen sections due to the lack of normal lung tissue in intraoperative frozen sections, and the remaining 294 cases were used to assess the agreement between the STAS diagnosis in frozen sections and paraffin sections. The flowchart of study design and inclusion/exclusion process is shown in Fig. 1.

### Clinical information

Clinical information included the following: (1) General information includes: gender, age, preoperative chest computed tomography (CT) and extent of surgical resection. CT features include: maximum tumor diameter (Tdmax) and consolidation-to-tumor ratio (CTR). Tdmax was defined as the maximum diameter of the tumor in the CT transverse section under the lung window (window width: 1600Hu, window center: -500Hu). CTR was defined as the ratio of solid components in mixed density ground glass nodules (GGN). GGN was defined as a limited increase in density without obscuring the bronchovascular bundle passing through it in lung window. CTR was equal to the maximum consolidation diameter divided by Tdmax. (2) Pathological information includes: diagnoses results on the intraoperative frozen sections and the postoperative paraffin sections.

### Determination of STAS

**Fig. 2:**
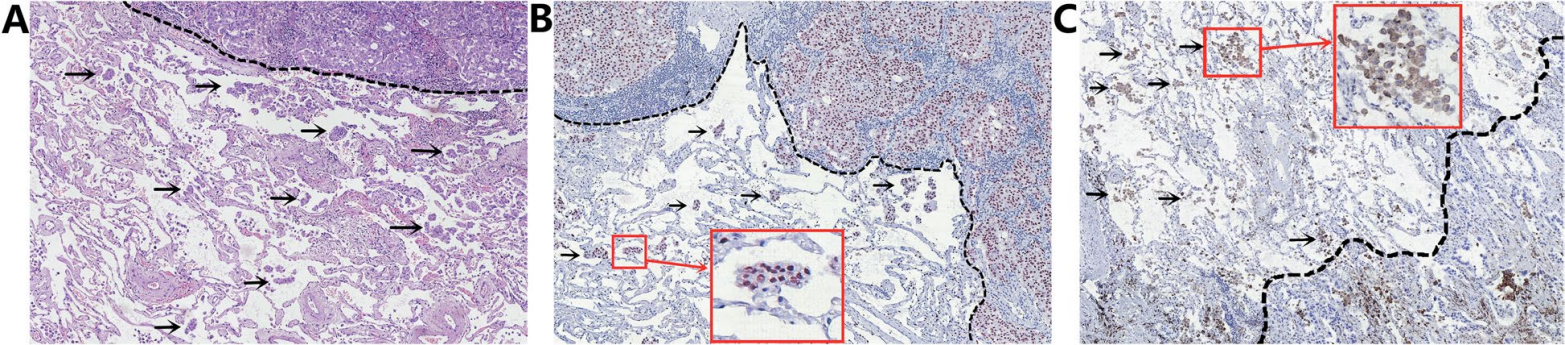
Pathological diagnosis of STAS. **A** Diagnosis of STAS by hematoxylin and eosin staining on paraffin sections. STAS (arrow) was located in the alveolar lumen outside the margin of the main tumor (black dashed line). **B** Immunohistochemical identification of STAS. STAS (arrow, TTF-1 positive) was located in the alveolar lumen outside the margin of the main tumor (black dashed line). **C** Immunohistochemistry identified non-tumor cells, mainly tumor-associated macrophages (arrows, CD68 positive). STAS: tumor spread through air spaces

Paraffin sections and frozen sections of surgical specimens from all enrolled patients were reviewed retrospectively, and the diagnoses of STAS on paraffin and frozen sections were recorded. Referring to the 2015 WHO lung cancer classification criteria and the study by Kadota et al. [3, 16], we defined STAS-positive as single tumor cells or clusters of tumor cells present in the alveolar lumen at least one alveolar septum away from the main tumor (Fig. 2A). In addition, the diagnosis of STAS on paraffin sections can be made with reference to immunohistochemistry (Fig. 2B, C) to distinguish other non-tumor cells (mainly tumor-associated macrophages). The sensitivity, specificity and accuracy of the diagnosis of STAS on intraoperative frozen sections were calculated using postoperative paraffin sections and immunohistochemistry as the gold standard for diagnosis of benign or malignant pulmonary nodules and STAS.

### Follow-up

Patients were followed up through postoperative visit records review and telephone calls. Overall survival (OS) was defined as the interval between the date of surgery and the date of death or last follow-up. Recurrence-free survival (RFS) was defined as the interval between the date of surgery and the date of the initial diagnosis of recurrence or last follow-up visit. Patients without an event were censored at the last follow-up date, September 2022.

### Statistical analysis

SPSS 25.0 software was used for the analysis. Measurement data were expressed as mean ± standard deviation (SD). Counting data were expressed as percentages (%) and comparisons between groups were made using the χ2 test or Fisher’s exact test. Concordance was tested using the Kappa test. *Kappa* (*K*) values were interpreted as follows [17]: below 0.4, low agreement; 0.4 to below 0.6, moderate agreement; 0.6 to below 0.8, substantial agreement; 0.8 to below 1.0, almost perfect. The Kaplan–Meier method was used to plot survival curves, and the Cox proportional hazard model was used to perform subgroup analysis for OS and RFS. *P* < 0.05 was considered a statistically significant difference.

## Results

### Clinical features

A total of 352 patients were included in this study. The rate of STAS-positivity on paraffin sections was 35.2% (124/352). 58 intraoperative frozen sections lacked the alveolar space outside the tumor to assess STAS. In the other 294 patients, 36.4% (107/294) cases were positive for STAS on paraffin sections, and 29.6% (87/294) were positive for STAS on frozen sections. The characteristics of all included patients are shown in Table 1.

**Table 1:** The characteristics of patients with small-sized clinical stage I NSCLC (*n* = 352)

Gender, n (%)	Male	161 (45.7)
	Female	191 (54.3)
Age (years), mean ± SD		60.97 ± 8.10
Tdmax (cm), mean ± SD		1.52 ± 0.38
CTR, mean ± SD		0.57 ± 0.36
Scope of surgery, n (%)	Lobectomy	307 (87.2)
	Sublobectomy	45 (12.8)
Pathological type, n (%)	Adenocarcinoma	318 (90.3)
	Squamous carcinoma	28 (8.0)
	Other	6 (1.7)
STAS on paraffin sections, n (%)	Positive	124 (35.2)
	Negative	228 (64.8)
STAS on frozen sections, n (%)	Positive	207 (58.8)
	Negative	87 (24.7)
	Unable to assess	58 (16.5)

*NSCLC* Non-small cell lung cancer, *SD* Standard deviation, *Tdmax* Maximum tumour diameter, *CTR* Consolidation-to-tumour ratio, *STAS* Tumor spread through air spaces

### Diagnostic performance of intraoperative frozen sections to detect invasive NSCLC

The accuracy of frozen sections for the diagnosis of invasive NSCLC in 58 patients whose frozen sections could not be evaluated for STAS and that in 294 patients whose frozen sections could be evaluated for STAS are 96.6% (56/58) and 95.2% (280/294) respectively, with no significant difference (*P* > 0.05), suggesting that frozen sections with alveolar spaces outside the main tumor for the diagnosis of STAS can also ensure the accuracy of the diagnosis of invasive NSCLC.

### Diagnostic performance of frozen sections to detect STAS

The diagnostic performance of frozen sections to detect STAS in 294 patients is shown in Table 2. The accuracy of frozen sections to diagnose STAS was 74.14% (218/294), sensitivity was 55.14% (59/107), specificity was 85.02% (159/187), and agreement was fair (*K* = 0.418). Patients were further divided into CTR ≤ 0.5 group (*n* = 140) and CTR > 0.5 group (*n* = 154), Tdmax ≤ 1 cm group (*n* = 53) and Tdmax > 1 cm group (*n* = 241) according to CT imaging characteristics for subgroup analysis of concordance. The *Kappa* value for the diagnosis of STAS on frozen sections was 0.368 for patients with CTR ≤ 0.5, with low agreement, while the *Kappa* value for patients with CTR > 0.5 was 0.415, with moderate agreement. In addition, the *Kappa* value for the diagnosis of STAS in frozen sections was 0.454 for patients with Tdmax ≤ 1 cm and 0.408 for patients with Tdmax > 1 cm, with moderate agreement in both subgroups.

**Table 2:** Diagnostic performance of frozen sections to detect STAS (*n* = 294)

Grouping	Diagnosis of STAS in frozen sections	Diagnosis of STAS in paraffin sections			
		Positive	Negative	Total	
All	Positive	59	28	87	Sensitivity: 55.14%
	Negative	48	159	207	Specificity: 85.02%
	Total	107	187	294	*Kappa* = 0.418 (moderate)
CTR ≤ 0.5	Positive	17	12	29	Sensitivity: 45.94%
	Negative	20	91	111	Specificity: 88.34%
	Total	37	103	140	*Kappa* = 0.368 (low)
CTR > 0.5	Positive	42	16	58	Sensitivity: 60.00%
	Negative	28	68	96	Specificity: 80.95%
	Total	70	84	154	*Kappa* = 0.415 (moderate)
Tdmax ≤ 1 cm	Positive	8	5	13	Sensitivity: 57.14%
	Negative	6	34	40	Specificity: 87.17%
	Total	14	39	53	*Kappa* = 0.454 (moderate)
Tdmax > 1 cm	Positive	51	23	74	Sensitivity: 54.83%
	Negative	42	125	167	Specificity: 84.45%
	Total	93	148	241	*Kappa* = 0.408 (moderate)

*STAS* Tumor spread through air spaces, *CTR* Consolidation tumor ratio, *Tdmax* Maximum tumour diameter

### Association of STAS on frozen sections and paraffin sections with prognosis

**Fig. 3:**
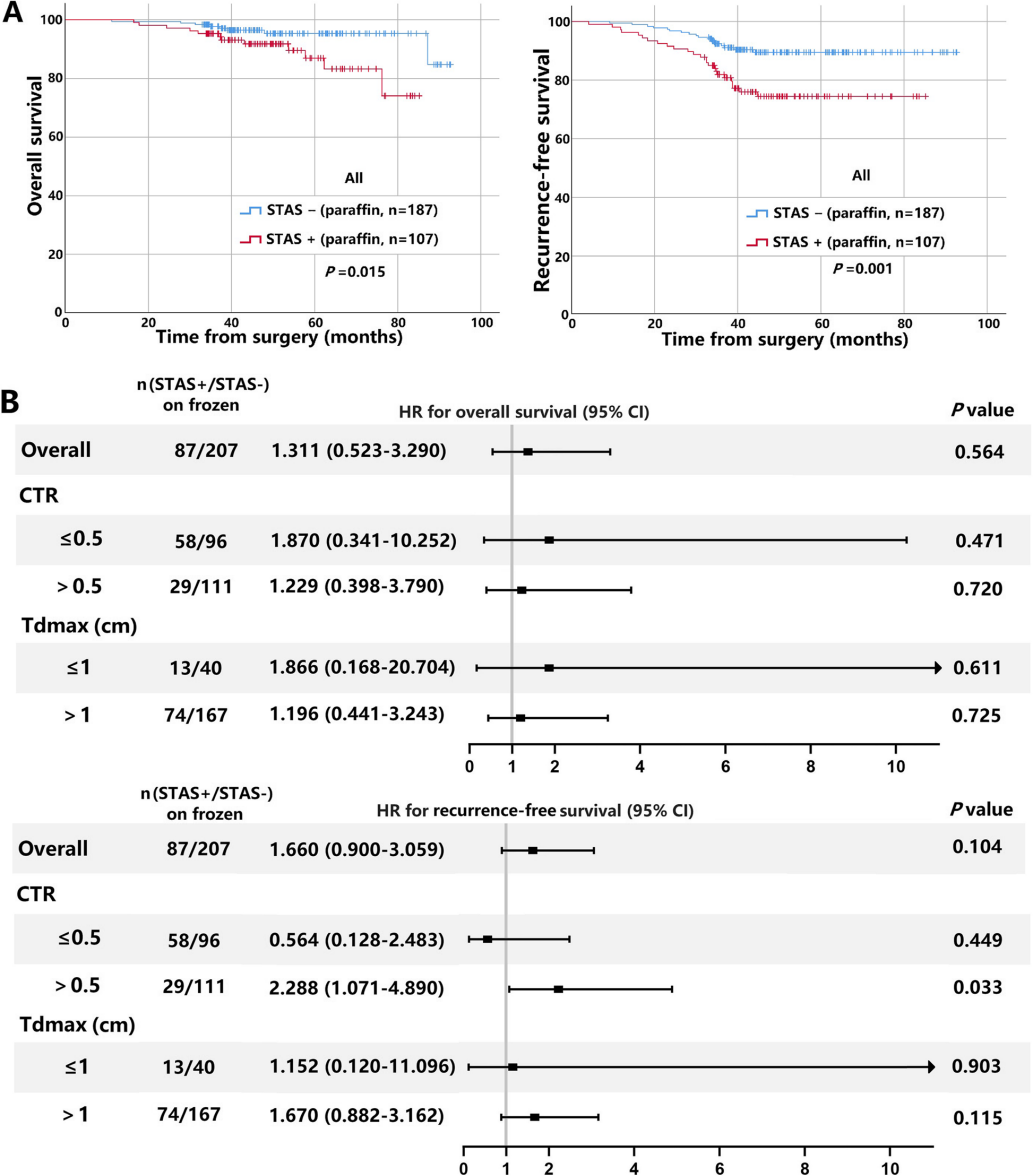
The impact of STAS on paraffin and frozen sections on the prognosis of clinical stage IA small-sized NSCLC. **A** Kaplan–Meier curves for OS (left panel) and RFS (right panel) curves of patients with STAS-positive and negative on paraffin sections. **B** Forest plots of OS (upper panel) and RFS (lower panel) of patients with STAS-positive and negative on frozen sections. STAS: tumor spread through air spaces; NSCLC: non-small cell lung cancer; OS: overall survival; RFS: recurrence-free survival; Tdmax: maximum tumour diameter; CTR: consolidation tumor ratio

The median follow-up time for the 294 patients was 48.1 months (range, 11.2–92.7 months). As shown in Fig. 3, RFS and OS were worse in patients with STAS-positive in paraffin sections than in STAS-negative ones (*P* < 0.05), whereas RFS and OS in patients with STAS-positive in frozen sections were not significantly different from those in STAS-negative ones (*P* > 0.05). In subgroup analysis, patients with STAS-positive on frozen sections had worse RFS than those with STAS-negative in CTR > 0.5 group (*P* < *0*.05). However, RFS and OS were not significantly different in patients with STAS-positive on frozen section compared with STAS-negative ones in Tdmax ≤ 1 cm group and Tdmax > 1 cm group (*P* > 0.05).

## Discussion

2021 WHO Classification of Thoracic Tumors explicitly identifies STAS as an aggressive histological feature of lung cancer with prognostic significance [2]. This study included clinical stage I invasive NSCLC ≤ 2 cm, and similarly showed that STAS-positive patients had significantly worse OS and RFS than STAS-negative patients (*P* < 0.05). Meanwhile, some previous studies have shown that patients with STAS-positive stage I NSCLC who underwent lobectomy have a better prognosis than those who underwent sublobectomy [7]. These findings suggest that more careful treatment of patients with STAS may be needed to improve the prognosis. However, there is still a lack of very accurate tools of diagnosing STAS preoperatively and intraoperatively, and frozen sections, as an important part of rapid intraoperative diagnosis, are certainly hold promise as a reliable means to diagnose STAS intraoperatively.

Although some scholars have questioned the presence of STAS in sections, suggesting that STAS may be the spread of tumor cell clusters caused by knife cuts during specimen processing [18], more and more studies have recently shown that STAS is an in vivo phenomenon rather than artifacts resulting from specimen handling procedures [19, 20]. At the same time, this artifact is mostly identifiable, mainly as mechanically separated tumor floaters or bands of tumor cells shed from the alveolar wall or pulmonary interstitium. But even so, there are still difficulties in the identification of STAS in intraoperative frozen sections. At present, the limitations of the diagnosis of STAS on frozen sections are mainly caused by section preparation. Morimoto et al. [21] suggested that the diagnosis of STAS on frozen sections is limited by the quality of sections, and that the insufficient expanded alveolar spaces in frozen sections may affect the microscopic observation of tumor cell clusters and further affect the frozen section diagnosis of STAS. Meanwhile, it is difficult to complete rapid immunohistochemical detection to distinguish tumor cells from other non-tumor cells in the alveolar spaces during intraoperative diagnosis. In this study, the diagnostic performance of the frozen section diagnosis of STAS in 294 intraoperative frozen sections where STAS could be assessed was evaluated using the paraffin sections diagnosis of STAS as the gold standard, yielding a sensitivity of 55.14%, a specificity of 85.22%, a *Kappa* value of 0.418 and moderate agreement. The slightly higher *Kappa* value compared to previous literature [11, 12] may be due to our inclusion of small-sized nodules (≤ 2 cm), which allows for the possibility of more adequate extra-tumoral alveolar cavities to visualize STAS in frozen sections, thus improving the accuracy of frozen section diagnosis of STAS, but the diagnostic accuracy is still unsatisfactory. Also, the result of survival analysis in this study indicated that STAS on paraffin sections was associated with worse prognosis OS and RFS (*P* < 0.05), consistent with previous literature reports [4, 22]. However, there was no significant difference in OS and RFS between patients with STAS-positive and negative frozen sections (*P* > 0.05), suggesting difficulties in identifying high-risk patients with poor prognosis through intraoperative frozen section diagnosis of STAS. It appears that there are still challenges in diagnosing STAS solely through intraoperative frozen sections and thus guiding the choice of surgical methods.

We further performed survival analysis and consistency test in different subgroups classified according to CTR and Tdmax. In this study, we found that the concordance between frozen and paraffin sections for STAS diagnosis was better in the CTR > 0.5 group than in the CTR ≤ 0.5 group (*K* = 0.415, moderate agreement vs. 0.368, low agreement). In the meantime, RFS was significantly lower in patients with STAS-positive frozen sections than in STAS-negative ones in the CTR > 0.5 group, suggesting that frozen section diagnosis of STAS helps to identify those at high risk of poor prognosis in small-sized (≤ 2 cm) clinical stage I NSCLC with CTR > 0.5. This may be related to the high incidence of STAS, widespread dissemination, and more micropapillary clustered dissemination in solid-dominant NSCLC [23, 24], and hence easier detection of STAS in frozen sections; and clustered dissemination of STAS is also easier to distinguish from non-tumor cells and exfoliated tumor cells due to knife cutting than the single-cell dissemination [16]. In addition, the margin of solid nodules is generally more clearly defined than GGN, which facilitates microscopic determination of tumor boundaries and may help to accurately identify STAS in frozen sections. Therefore, we believe that intraoperative frozen section diagnosis of STAS is of interest in patients with small-sized NSCLC with CTR > 0.5.

**Fig. 4:**
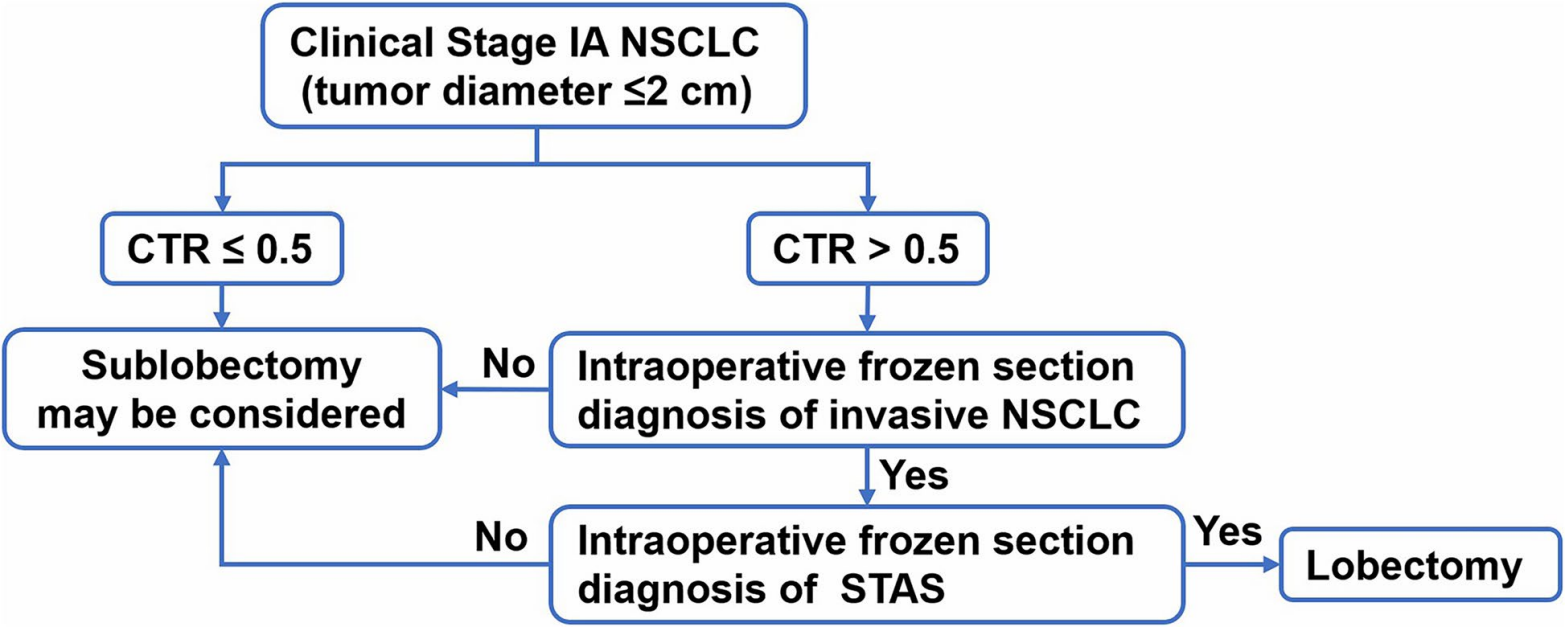
Suggested surgical procedure for clinical stage I small-sized NSCLC (≤ 2 cm) based on the frozen section diagnosis of STAS. NSCLC: non-small cell lung cancer; CTR: consolidation tumor ratio; STAS: tumor spread through air spaces

It is worth noting that the results of the recent JCOG0802/WJOG4607L study suggest that segmentectomy should be the standard procedure for clinical stage IA small-sized (tumor diameter ≤ 2 cm, CTR > 0.5) NSCLC, as this study was designed relatively early and did not adequately consider the impact of STAS [10]. Conversely, many studies support lobectomy in patients with STAS-positive stage I NSCLC [7–9, 16]. In addition, Ren et al. [25] also found STAS in residual lung segments of simulated sublobectomy. Therefore, we generally support the results of the JCOG0802/WJOG4607L study, but in the context of this study we recommend intraoperative frozen sections examination of STAS for patients who undergo sublobectomy with CTR > 0.5 to determine whether to proceed with lobectomy. Referring to the suggested surgical approach by STAS positivity in NSCLC proposed by Toki et al. [9], we propose a reference procedure for the selection of surgical treatment modality for clinical stage I NSCLC (≤ 2 cm) based on intraoperative frozen section diagnosis for STAS (Fig. 4). Meanwhile, we need to further improve the quality of frozen sections or combine them with other effective and rapid examination as to improve the intraoperative identification of STAS and avoid irreversible and unnecessary radical surgery due to false-positive results [26].

This study has certain limitations: (1) This study is a retrospective study, and it is difficult to avoid selection bias. Therefore, further prospective validation studies are needed. (2) There were no uniform standards for producing intraoperative frozen sections in the past, and some frozen sections had few or even no alveolar lumen outside the main tumor, which affected the accuracy of STAS assessment on frozen sections, and may even fail to assess STAS. Besides, most of the previous frozen sections had mild discoloration, which would also affect our analysis of the accuracy of STAS assessment on actual frozen sections. Therefore, prospective studies are needed to develop uniform and appropriate frozen section criteria to accurately identify STAS in intraoperative frozen sections. (3) Although data from two centres were included in this study, the sample size is still limited and studies with more centers and larger cases are needed. (4) This study mainly focused on STAS, but other pathological features such as tumor grade, vessel invasion, pleural invasion, etc., may also affect intraoperative treatment decisions. We will further combine these indicators effectively in order to more accurately identify patients with poor prognosis intraoperatively and select appropriate surgical approach.

## Conclusion

In summary, the diagnostic performance of frozen sections for STAS in clinical stage I NSCLC (≤ 2 cm) is limited, but the accuracy of frozen section diagnosis for STAS in small-sized NSCLC with CTR > 0.5 is higher than those with CTR ≤ 0.5 and has prognostic significance. This may inform surgeons in making intraoperative decisions about treatment options for clinical stage I NSCLC (tumor diameter ≤ 2 cm; CTR > 0.5). We expect that future improvements in producing intraoperative frozen sections and better diagnostic techniques will lead to more accurate identification of STAS.

## Data Availability

The datasets used during the current study are available from the corresponding author upon reasonable request.
